# Context matters: reconsidering resilience factors in dehumanization pathways to dysfunctional eating among transgender and gender diverse individuals

**DOI:** 10.3389/fpsyt.2026.1788398

**Published:** 2026-03-23

**Authors:** Marina Bonato, Benedetta Cherchi, Paolo Meneguzzo, Lucia Ronconi, Alberto Scala, Benedetta Tascini, Michela Gatta, Andrea Garolla, Marina Miscioscia

**Affiliations:** 1 Department of Developmental Psychology and Socialization, University of Padova, Padova, Italy; 2 Regional Reference Center for Gender Incongruence (CRRIG), University Hospital of Padova, Padova, Italy; 3 Unit of Andrology and Reproductive Medicine, Department of Medicine, University of Padova, Padova, Italy; 4 Department of Neuroscience, University of Padova, Padova, Italy; 5 Computer and Statistical Services, Multifunctional Pole of Psychology, University of Padua, Padua, Italy; 6 Child and Adolescent Neuropsychiatry Unit, Department of Women’s and Children’s Health, University of Padova, Padova, Italy

**Keywords:** eating disorders, pantheoretical model of dehumanization, resilience factors, transgender and gender diverse, TRIM model

## Abstract

**Introduction:**

Transgender and gender diverse (TGD) individuals experience disproportionately high rates of disordered eating (DE), yet little is known about how resilience factors operate within risk pathways linked to dehumanization. Drawing on the Pantheoretical Model of Dehumanization (PMD) and the Transgender Resilience Intervention Model (TRIM), this study examines whether individual- and group-level resilience factors moderate associations between dehumanization, internalization of beauty standards (IBS), and DE among TGD adults.

**Methods:**

Between November 2024 and May 2025, a sample of 122 TGD individuals completed validated measures assessing minority stress and resilience (Gender Minority Stress and Resilience Measure), sexual objectification (Interpersonal Sexual Objectification Scale), IBS (Sociocultural Attitudes Toward Appearance Questionnaire - Social Media), DE symptoms (Eating Disorder Examination - Questionnaire), and psychosocial resilience factors (Comprehensive Hope State Scale, Rosenberg Self-Esteem Scale, Transgender Identity Survey, and Multidimensional Scale of Perceived Social Support).

**Results:**

Exploratory moderated mediation analyses revealed several resilience factors (self-esteem, trust, self-realization, TGD pride, acceptance of TGD identity and acceptance of TGD expression) were associated with lower IBS and reduced DE symptoms. However, some factors also showed context-dependent patterns. TGD pride appeared to intensify the dehumanization-IBS association, while community belonging and identity acceptance were associated with amplified links between IBS and DE outcomes, potentially suggesting that intracommunity appearance norms may reinforce cisnormative aesthetic pressures. Conditional indirect effects varied across levels of resilience factors, though the exploratory nature of these analyses warrants cautious interpretation.

**Discussion:**

These exploratory findings suggest that psychosocial resources may both mitigate and inadvertently intensify vulnerability to body-related distress, depending on sociocultural and community contexts. These findings underscore the potential value of individualized clinical and community interventions that address the heterogeneous needs and lived experiences of TGD people.

## 1 Introduction

Eating disorders are disproportionately prevalent among transgender and gender diverse (TGD) individuals: those whose gender identity does not align with their sex assigned at birth (1). Compared with cisgender peers, TGD individuals face a two- to fourfold higher risk (2–5), commonly attributed to gender-related body incongruence and body image dissatisfaction (6–9). These experiences frequently give rise to maladaptive weight- and shape-control behaviours (2, 10–12).

The Pantheoretical Model of Dehumanization (PMD) conceptualizes dehumanization as the combined action of discrimination and sexual objectification, two interrelated processes that intensify body image disturbances and disordered eating (DE) (13). TGD people are persistently exposed to anti-transgender discrimination rooted in heterosexist social structures (14, 15), while simultaneously experiencing sexual objectification (16), as their bodies are routinely subjected to sexualizing gazes in everyday interactions (17, 18) and degrading or stereotyped media portrayals (19, 20). Together, these stressors operate as dual pathways of dehumanization, fostering shame, hypervigilance, body surveillance and pressure to conform to hypergendered aesthetic norms (21–23).

These processes also reinforce the internalization of sociocultural beauty standards (IBS), a key mediator linking dehumanization to body monitoring, body dissatisfaction, and DE (21). Such standards include thinness, vulnerability, and normative femininity among transgender women, as well as muscularity, dominance, and hypermasculinity among transgender men (16, 24–28). The PMD has been tested among both transgender women (22) and transgender men (23), however, it has not yet been examined among gender-diverse (GD) individuals, despite emerging evidence that GD people are similarly vulnerable to objectification and dehumanization (29).

The role of resilience factors in the pathways leading to DE among TGD individuals remains markedly underexplored. Existing evidence suggests potential benefits: social support has been inversely associated with DE in TGD youth (30), self-esteem and life satisfaction have been shown to fully mediate the relationship between harassment, rejection, and body appreciation (31), highlighting their potential buffering role. However, resilience factors widely assumed to be uniformly beneficial for TGD people appear to have context-dependent effects.

Siconolfi et al. (32) found a positive association between LGBTQ+ community affiliation and body dissatisfaction, indicating that community connection is not always protective. This pattern is reinforced by research on gay community stress, which shows that community involvement can be associated with higher body dissatisfaction (33–36). Simeone et al. (37) describe the queer community as simultaneously body-positive and characterized by restrictive appearance ideals to avoid queer-related rejection (38).

As noted by Santoniccolo et al. (39), intraminority stress may heighten appearance-based comparisons, reduce body satisfaction, and increase body surveillance. At the same time, other studies identify community connectedness as a factor that may moderate the effects of body dissatisfaction and support psychological well-being (40). These findings suggest that community involvement may be protective or harmful depending on the specific sociocultural context.

Moreover, TGD individuals often interpret anti-transgender discrimination and violence as resulting from bodies not yet seen as aligned with their affirmed gender (39). This attribution can be context-dependent: in supportive environments, motivation to increase gender congruence may foster identity affirmation, a recognized resilience factor within the Transgender Resilience Intervention Model (TRIM) (41). However, in more hostile or objectifying contexts, the same motivation can intensify appearance surveillance and reinforce pressure to conform to hypergendered aesthetic norms. Guilt-related cognitions, may further complicate these dynamics by driving compensatory eating behaviours and contributing to interpersonal distrust (42). Such dynamics may drive efforts to attain body shapes culturally associated with one’s affirmed gender, thereby increasing vulnerability to body dissatisfaction and DE behaviours.

Thus, resilience factors in TGD people may not function as uniformly buffering resources, rather, their effects can become risk-amplifying as a function of context. Greater identity affirmation and community involvement may increase visibility and, consequently, exposure to objectification, microaggressions, and appearance-based scrutiny, particularly within hostile hetero-cisnormative environments. Moreover, intracommunity norms may inadvertently reproduce cisnormative aesthetic standards by privileging “passable” bodies and transition narratives, thereby intensifying appearance-based social comparison and social media benchmarking. Under such conditions, resources that typically promote well-being may simultaneously strengthen specific risk pathways, reinforcing IBS and, in turn, dysfunctional eating outcomes.

We operationalize “context” through: (1) social media-based internalization of beauty standards (SATAQ-SM), capturing exposure to cisnormative aesthetic ideals; (2) community connectedness and TGD pride, reflecting embeddedness in TGD social networks; (3) reported sources of gender norm pressure (family, workplace, society, LGBTQ+ community, social media); and (4) dehumanization experiences (discrimination and objectification).

**Figure 1:**
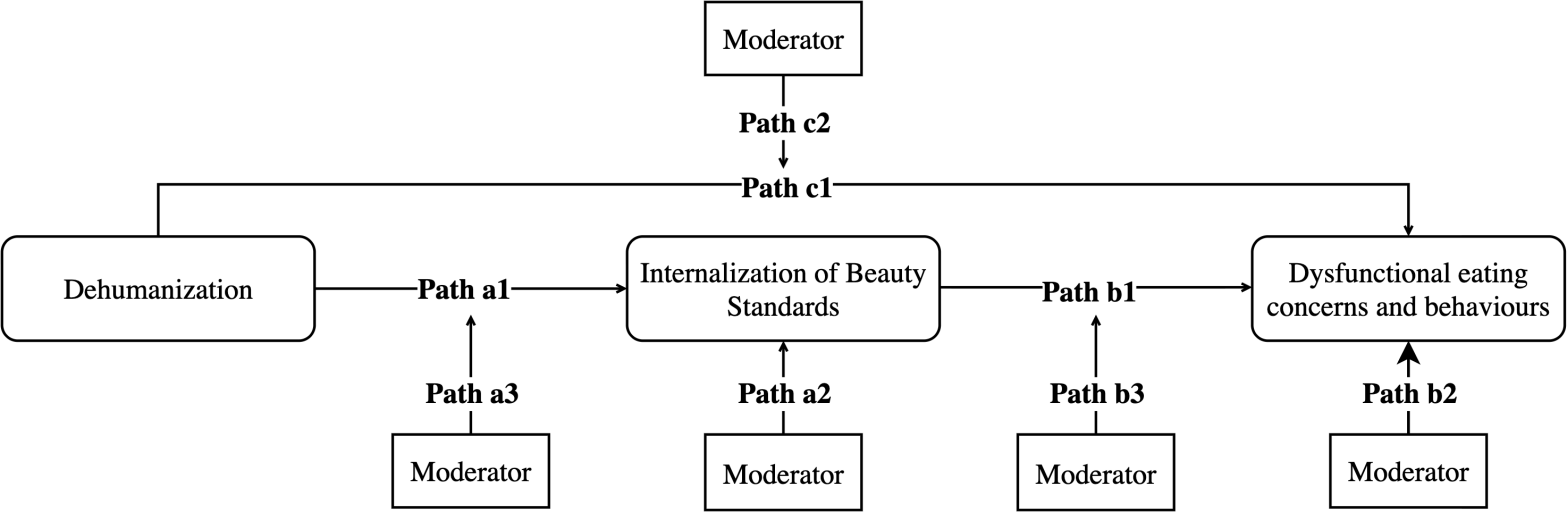
Conceptual model of the moderated effects of resilience factors on the pathway from dehumanization composite index to dysfunctional eating concerns and behaviours via IBS.

Guided by the PMD and TRIM, we propose that psychosocial resilience factors may shape this process at multiple points: Path A (Dehumanization composite index → IBS), Path B (IBS → eating-related outcomes), and Path C (Dehumanization composite index → eating-related outcomes) (see **Figure 1**). This framework allows us to test whether resilience factors function as buffers or as context-dependent amplifiers. A secondary aim is to assess whether GD individuals also exhibit pathways in which dehumanization predicts DE through the IBS. We tested four hypotheses regarding resilience factors. H1: Individual- and group-level resilience factors would be directly associated with lower IBS and lower eating-related outcomes, consistent with a general resilience function. H2: Resilience factors would moderate the association between dehumanization composite index and IBS. H3: Resilience factors would moderate the association between IBS and eating-related outcomes. H4: Resilience factors would moderate the direct association between dehumanization composite index and eating-related outcomes. We define a resilience resource as protective only when it demonstrates an empirically observed risk-reducing effect in the tested models. Additionally, we examined whether the PMD model would be supported in the GD population. Specifically, we hypothesized that IBS would mediate the association between dehumanization composite index and eating-related outcomes (shape concern, weight concern, eating concern, and restraint behaviour).

## 2 Materials and methods

### 2.1 Participants and procedures

Ethical approval was obtained from the Territorial Ethics Committee (Area CEV-6011/AO/24) and the Ethical Committee of Psychological Research, Area 17, at the University of Padova (Approval No. 794-a). Informed consent was obtained from all participants prior to participation. Data were collected online (November 2024 - May 2025). Eligibility criteria required participants to self-identify as TGD, be at least 18 years old, and have initiated their social or psychological, or medical Gender Affirmation (GA). Recruitment occurred in person at the Regional Reference Center for Gender Incongruence in Padova, via social media, flyers, and snowball sampling. The Qualtrics questionnaire, accessible via QR code or link, required about 35 minutes to complete.

### 2.2 Measures

Participants reported demographics including age (in years), gender identity (man, woman, nonbinary, genderqueer), ethnic background (caucasian, black, asian, hispanic/latinx, indigenous), education (primary school to PhD), employment (full-time, part-time, unemployed, student, working student, retired), and marital status (not married, married/civil union, separated, divorced, widowed). They indicated the age at which they began exploring their gender identity (<12, 13-17, 18-24, 25-34, 35-44, 45+ years) and their interest in Gender-Affirming Hormone Therapy (GAHT) (do not wish to start, unsure, wish to start but have not yet had the opportunity, already started). Participants reported the duration of their GA (<1, 1-2, 3-5, 6-10, >10 years).

They indicated how often they felt pressure to conform to gender norms (5-point likert scale) and its sources (family, friends, workplace/school, broader society, LGBTQ+ community, social media). They reported how this pressure influenced their GA (positively, no impact, caused confusion/uncertainty, negatively), and selected their motivations for beginning their GA (alleviate gender dysphoria; affirm gender identity; improve mental health/well-being; increase social acceptance; physical comfort; personal satisfaction; relational satisfaction; pressure to conform to societal gender norms). They also indicated whether anticipated social acceptance played an important, minor, or no role in their decision, and rated how important initiating their GA had been for their overall well-being (5-point likert scale). “Other” and “prefer not to say” options were available.

Experiences of anti-transgender discrimination and minority stress were assessed with the 56-item Gender Minority Stress and Resilience Measure (GMSR) (43), with higher scores indicating greater minority stress. Sexual objectification was measured using the 30 items Interpersonal Sexual Objectification Scale (ISOS) (44), with higher scores indicating more frequent experiences of sexual objectification. As no Italian ISOS was available, an Italian adaptation was developed following standard procedures (45).

IBS ideals were measured with the Sociocultural Attitudes Toward Appearance Questionnaire - Social Media (SATAQ-SM) (46). The scale consists of 30 items with higher scores reflecting stronger IBS. DE concerns and behaviours were assessed using the 28-item Eating Disorder Examination-Questionnaire (EDE-Q) (47). Higher scores represent greater severity of DE symptoms.

Individual and group-level resilience factors were assessed using the Comprehensive Hope State Scale (CHS-S) (48, 49), which includes 37 items. Higher scores reflect greater current levels of hope. Additional individual resources were measured with the 10-item Rosenberg Self-Esteem Scale (RSES) (50), where higher scores indicate greater self-esteem, and the GMSR Resilience subscale, with higher scores reflecting greater levels of resilience-related factors.

Acceptance of one’s gender identity, gender expression, and sense of community was assessed using the Shame, Passing, and Alienation subscales of the Transgender Identity Survey (TIS) (51), reverse-coded so that higher Shame scores indicate greater acceptance of one’s gender identity, higher Passing scores denote greater acceptance of one’s gender expression, and higher Alienation scores indicate a greater sense of community. Perceived social support was measured using the 12-item Multidimensional Scale of Perceived Social Support (MSPSS) (52). Higher scores indicate stronger perceived social support.

### 2.3 Data preparation and statistical analyses

Scores from all psychometric subscales were standardized into z-scores. Age was grouped into three ordinal brackets (1 = 18-24, 2 = 25-34, 3 = 35-64), while Body Mass Index (BMI) was mean-centered and treated as a continuous variable. Participants were grouped into three gender identity categories: transgender women, transgender men, and gender-diverse individuals. The gender-diverse group included all participants who identified with non-binary or other gender-diverse labels.

Missing values were below 5% for the ISOS, EDE-Q, and CHS subscales, and below 10% for the TIS subscales. Missing data were handled using Predictive Mean Matching (PMM) with *m =* 20 imputed datasets (20 interactions) for multiple regression analyses and Full Information Maximum Likelihood (FIML) for all path models.

A composite z-standardized Dehumanization index, consistent with PMD theory, was calculated as the mean of two variables: experiences of Body Evaluation in the past year and anti-transgender discrimination. We selected Body Evaluation (past year) because it is the component of objectification that increases body surveillance and pressure to meet normative beauty standards, contributing to disordered eating. We used the past-year timeframe because objectification experiences can change substantially during gender affirmation (e.g., changes in visibility and how others perceive the body). A recent window therefore provides a more comparable indicator across participants at different stages of affirmation, whereas lifetime measures would mix pre- and post-affirmation periods and be less comparable across individuals. This composite reflects the combined burden of identity-based discrimination and appearance-based objectification, two pathways through which TGD people experience dehumanization.

Statistical analyses were conducted using R (version 4.4.3) (53). The psych package (version 2.4.6.26) (54) was used for descriptive and correlational analyses, and the lavaan package (version 0.6-18) (55) was used to estimate regression-based path models.

Multiple linear regression analyses were used to examine the simultaneous effects of relevant covariates (age, BMI, time since GA, and gender identity) on each psychometric outcome, allowing us to control for potential confounding variables across 31 psychometric scales. These models used pooled estimates from the 20 imputed datasets following Rubin’s rules.

Prior to testing moderation, we estimated simple mediation models to establish the baseline mediated pathway: Dehumanization composite index → IBS → ED outcomes. These models were estimated separately for each of the four ED outcomes. Covariates (age, BMI, time since GA and gender identity) were included in all path models. All simple mediation analyses were replicated within the GD subgroup. Covariates (age, BMI, and time since GA) were included.

To test the moderating effects of individual- and group-level resilience factors, we estimated separate moderated mediation models for each resilience factor × outcome combination (13 moderators × 4 outcomes = 52 total models). Each model tested one resilience factor as a potential moderator of Paths A (Dehumanization composite index → IBS), B (IBS → outcome), and C (Dehumanization composite index → outcome). All moderators were mean-centered prior to computing interaction terms. Conditional indirect effects were estimated at the 16th, 50th, and 84th percentiles of each moderator’s distribution (approximating −1 SD, mean, and +1 SD for near-normal distributions). Covariates (age, BMI, time since gender affirmation, and gender identity) were included in all path models. All path models were estimated using Maximum Likelihood (ML). All models used 5,000 bootstrap resamples with bias-corrected and accelerated (BCa) confidence intervals to estimate indirect and conditional indirect effects.

### 2.4 Power analysis

The minimum required sample size was estimated *a priori* using G*Power 3.1 for a multiple linear regression analysis with a moderate effect size (f² = .15), α = .05, power (1-β) = .90, and 5 predictors. The resulting minimum sample size was 116 participants. Because all variables in our path analysis were observed, the structural model is mathematically equivalent to a set of multiple regression equations.

## 3 Results

### 3.1 Participant characteristics

A total of 136 individuals participated in the study. 14 incomplete responses were excluded, yielding a final sample of 122 participants aged 18 and 60 years (M = 25.9, SD = 7.59, Mdn = 24). Demographic characteristics of the sample are reported in **Table 1** and descriptive statistics for all psychometric measures in **Table 2**.

**Table 1:** Demographic characteristics of the sample.

Age range	n(%)		
1	65	(53.3)	
2	45	(36.9)	
3	8	(6.6)	
4	2	(1.6)	
5	2	(1.6)	
Gender Identity			
Genderqueer	12	(9.8)	
Man	55	(45.1)	
Non-binary	25	(20.5)	
Prefer not to say	5	(4.1)	
Woman	25	(20.5)	
Ethnic Background			
Asian	1	(0.8)	
Hispanic/Latinx	4	(3.3)	
Other	2	(1.6)	
Caucasian	114	(93.4)	
Caucasian, Indigenous	1	(0.8)	
BMI range			
15,1-23,2	67	(54.9)	
23,3-31,4	42	(34.4)	
31,5-39,6	10	(8.2)	
39,7-47,8	2	(1.6)	
47,9-56,1	1	(0.8)	
BMI Range Number			
1	67	(54.9)	
2	42	(34.4)	
3	10	(8.2)	
4	2	(1.6)	
5	1	(0.8)	
Education			
Bachelor Degree	36	(29.5)	
High school	61	(50.0)	
Master Degree	15	(12.3)	
Middle school	10	(8.2)	
Employment Status			
Full-time employee	25	(20.5)	
Part-time employee	21	(17.2)	
Prefer not to say	3	(2.5)	
Student	39	(32.0)	
Student worker	23	(18.9)	
Unemployed	11	(9.0)	
Marital Status			
Married or in a civil union	4	(3.3)	
Not married	109	(89.3)	
Other	1	(0.8)	
Prefer not to say	8	(6.6)	
Age of Gender Identity Exploration			
Before age 12	21	(17.4)	
13–17 years	57	(47.1)	
18–24 years	33	(27.3)	
25–34 years	7	(5.8)	
35–44 years	2	(1.7)	
Prefer not to say	1	(0.8)	
Interest in GAHT			
I am not sure if I want to start GAHT	12	(9.8)	
I do not wish to start GAHT	9	(7.4)	
I have started GAHT	68	(55.7)	
I prefer not to say	1	(0.8)	
I would like to start GAHT, but I have not yet had the opportunity	32	(26.2)	
Duration of Gender Affirmation Path			
Less than 1 year	16	(13.3)	
1–2 years	21	(17.5)	
3–5 years	56	(46.7)	
6–10 years	18	(15.0)	
More then 10 years	5	(4.2)	
Prefer not to say	4	(3.3)	
Gender Affirmation Duration Range			
1	16		(13.1)
2	21		(17.2)
3	62		(50.8)
4	18		(14.8)
5	5		(4.1)
Pressure to Conform Gender Norms			
Never	1		(0.8)
Rarely	8		(6.6)
Sometimes	31		(25.6)
Often	44		(36.4)
Very often	37		(30.6)
Source of Gender Pressure			
Pressure Family	35		(28.9)
Pressure School Work	33		(27.3)
Pressure Society	63		(52.1)
Pressure Social Media	11		(9.1)
Pressure LGBTQ+ Community	7		(5.8)
Pressure Friends	5		(4.1)
Pressure Self	3		(2.5)
Pressure Healthcare	1		(0.8)
Impact of Gender Norm Pressure			
It caused confusion or uncertainty	76		(62.8)
It had no impact on my decisions	26		(21.5)
It negatively influenced my decisions	12		(9.9)
It positively influenced my decisions	7		(5.8)
Primary Motivation Gender Affirmation			
To alleviate gender dysphoria	77		(64.2)
To affirm my gender identity	67		(55.8)
To improve my mental health and well-being	68		(56.7)
For personal satisfaction	35		(29.2)
For relational satisfaction	13		(10.8)
For physical comfort	36		(30.0)
To increase social acceptance	14		(11.7)
Pressure to conform to societal gender norms	6		(5.0)
I have not started yet	1		(0.8)
Other	1		(0.8)
Role Social Acceptance			
No	44		(36.4)
Yes, a major role	34		(28.1)
Yes, a minor role	43		(35.5)
Importance Affirmation for Wellbeing			
Very important	102		(85.0)
Moderately important	13		(10.8)
Neutral	2		(1.7)
Prefer not to say	3		(2.5)
Very important	102		(85.0)

N, 122. The n column reports the number of participants per category, with percentages shown in parentheses.

**Table 2:** Descriptive statistics.

Psychometric variables	Missing	Mean	SD
Antitransgender discrimination	0	2.43	1.356
Rejection	0	2.21	1.521
Victimization	0	1.76	1.466
Non-affirmation	0	14.30	7.446
Internalized Transphobia	0	11.54	8.262
Pride	0	18.49	6.483
Negative Expectations	0	19.18	7.626
Non-disclosure	0	9.96	5.187
Community connection	0	12.86	4.511
Experiences of Body Evaluation across lifetime	1	2.53	0.888
Unwanted Explicit Sexual Advances across lifetime	1	1.98	0.853
Experiences of Body Evaluation in the past year	1	2.06	0.803
Unwanted Explicit Sexual Advances in the past year	1	1.38	0.612
Appearance Pressure	0	21.21	7.562
Internalization of Beauty Standards	0	24.40	9.147
Internalization of Athletic Ideals	0	13.53	4.986
Weight Concern	5	12.32	3.832
Shape Concern	2	3.23	1.747
Eating Concern	0	1.77	1.688
Restraint Behaviour	0	1.68	1.843
Support from Significant others	0	5.83	1.466
Family Support	0	4.05	1.887
Friends Support	0	5.84	1.274
Self-esteem	1	15.60	6.803
Trust	1	18.93	6.299
Spirituality	1	4.62	6.994
Self-Realization	1	10.20	4.898
Relationship and Social Support	1	24.40	8.757
Acceptance of one’s Gender Identity	10	4.66	1.632
Acceptance of one’s Gender Expression	10	3.86	1.469
Sense of Community	10	4.49	1.402

N, 122. Values represent missing values, sample means (M) and standard deviations (SD).

### 3.2 Psychometric evaluation of the ISOS past year and lifetime scales

Since the ISOS questionnaire was translated for the present study, the psychometric properties of the Past Year and Lifetime scales were examined through internal consistency analysis and exploratory factor analysis (EFA). Both scales demonstrated high internal consistency (α = .917 for Past Year; α = .946 for Lifetime). EFA indicated a three-factor structure for each scale, accounting for 52.5% and 58.2% of the total variance, respectively (Past Year: KMO = .868; Lifetime: KMO = .917; Bartlett’s test significant for both, p < 0.001). These preliminary findings support the reliability of the Italian ISOS in this sample. Confirmatory testing of this structure will be deferred to future work in a larger, independent validation sample.

### 3.3 Associations among study variables and extension of the PMD to gender-diverse individuals

**Figure 2:**
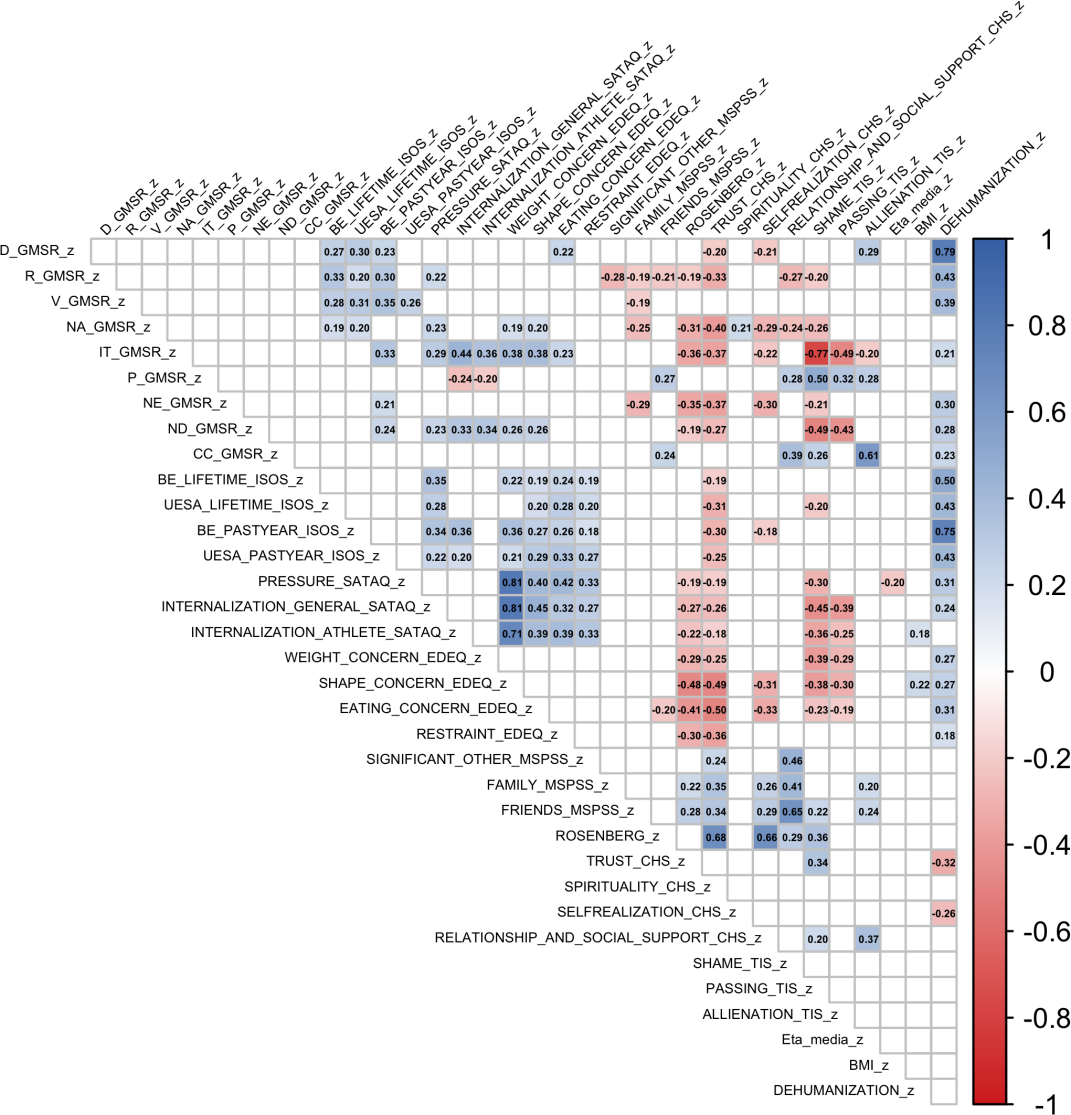
Spearman’s correlations heatmap N, 122. Heatmap showing Spearman’s correlations among standardized study variables. Only statistically significant correlations (*p* < 0.05) are displayed; Color intensity reflects strength and direction, with positive associations shown in blue and negative associations shown in red.

Multiple linear regression analyses were conducted to examine associations between sociodemographic variables and psychological measures. Full results are reported in **Table 3**. Spearman bivariate correlations were performed to evaluate the relationships among all study variables (**Figure 2**). Using a path analysis, we confirmed in our sample, consistent with Velez et al. (23) and Brewster et al. (22), that IBS mediates the association between dehumanization composite index and eating outcomes (**Supplementary Table 1**). We conducted a preliminary test of the same models within the subgroup of GD individuals (n = 42). The results, presented in **Table 4**, provide preliminary support for the model’s applicability to this group.

**Table 3:** Associations between sociodemographic variables and psychological measures with explained variance (R²).

Dependent variable	Gender identity: GenderDiverse	Gender identity: Man	Affirmation time	Age range	BMI range	R²; F
Non-affirmation	0.573*	—	-0.380***	—	0.037**	R² = .294; F(5, 109) = 9.07***
Internalized transphobia	—	—	-0.294**	—	—	R² = .123; F(5, 109) = 3.05*
Pride	—	-0.642**	—	—	—	R² = .119; F(5, 109) = 2.95*
Non disclosure	—	—	-0.228*	—	—	R² = .103; F(5, 109) = 2.51*
Experiences of Body Evaluation across lifetime	0.599*	—	—	—	—	R² = .051; F(5, 109) = 1.16
Unwanted Explicit Sexual Advances across lifetime	0.713**	—	—	—	—	R² = .087; F(5, 109) = 2.07
Experiences of Body Evaluationin the past year	-0.740**	-0.991***	—	—	—	R² = .160; F(5, 109) = 4.13**
Appearance pressure	—	—	—	—	0.032*	R² = .106; F(5, 109) = 2.59*
Internalization of beauty standards	—	—	-0.237*	—	—	R² = .112; F(5, 109) = 2.74*
Internalization of athletic ideals	—	—	-0.292**	—	—	R² = .145; F(5, 109) = 3.70**
Weight concern	—	—	-0.278**	—	—	R² = .139; F(5, 109) = 3.50**
Shape concern	—	—	-0.244**	—	0.038*	R² = .154; F(5, 108) = 3.95**
Support from significant others	—	0.641**	—	—	-0.040**	R² = .114; F(5, 109) = 2.82*
Self-esteem	—	—	0.255**	—	—	R² = .095; F(5, 109) = 2.29
Trust	—	—	0.219*	—	—	R² = .101; F(5, 109) = 2.43*
Self-realization	—	—	0.213*	—	—	R² = .068; F(5, 108) = 1.57
Acceptance of one’s gender expression	1.028***	—	—	—	—	R² = .196; F(5, 82) = 3.99**

N, 122. This table presents the results of multiple linear regression analyses examining the associations between sociodemographic predictors (gender identity, time since gender affirmation, age, and BMI range) and psychological variables. Only standardized coefficients (β) for significant predictors are reported with significant association marked as follow: *p <.05, **p <.01, ***p <.001. The final column reports the overall model fit, including the coefficient of determination (R²) and F-statistics for each regression. Missing data handled via Predictive Mean Matching (20 imputations, Rubin’s rules pooling). All VIF values < 1.82 (no multicollinearity).

**Table 4:** Path analysis testing IBS as a mediator between dehumanization composite index and eating-related outcomes among gender-diverse and non-binary individuals.

Path	Effect	β	SE	z	p	95% BCa CI
Shape Concern						
Dehumanization composite index → IBS (a)	Direct	0.595	0.136	4.374	<.001	[0.336, 0.883]
IBS → Shape Concern (b)	Direct	0.510	0.164	3.099	.002	[0.186, 0.814]
Dehumanization composite index → Shape Concern (c’)	Direct	0.201	0.187	1.075	.282	[-0.115, 0.649]
Dehumanization composite index → Shape Concern	Indirect	0.303	0.115	2.636	.008	[0.120, 0.567]
Dehumanization composite index → Shape Concern	Total	0.504	0.165	3.056	.002	[0.218, 0.883]
	Explained variance	IBS R² 0.294 Shape Concern R² 0.425				
Weight Concern						
Dehumanization composite index → IBS (a)	Direct	0.595	0.136	4.373	<.001	[0.336, 0.883]
IBS → Weight Concern (b)	Direct	0.815	0.096	8.511	<.001	[0.613, 0.988]
Dehumanization composite index → Weight Concern (c’)	Direct	0.162	0.100	1.627	.104	[-0.020, 0.371]
Dehumanization composite index → Weight Concern	Indirect	0.485***	0.117	4.162	<.001	[0.275, 0.740]
Dehumanization composite index → Weight Concern	Total	0.647	0.140	4.609	<.001	[0.377, 0.930]
	Explained variance	IBS R² 0.294 Weight Concern R² 0.821				
Eating Concern						
Dehumanization composite index → IBS (a)	Direct	0.598	0.135	4.438	<.001	[0.342, 0.885]
IBS → Eating Concerns (b)	Direct	0.412	0.214	1.930	.054	[0.009, 0.866]
Dehumanization composite index → Eating Concerns (c’)	Direct	0.259	0.229	1.132	.258	[-0.152, 0.759]
Dehumanization composite index → Eating Concerns	Indirect	0.247	0.138	1.785	.074	[0.029, 0.606]
Dehumanization composite index → Eating Concerns	Total	0.506	0.185	2.730	.006	[0.182, 0.917]
	Explained variance	IBS R² 0.299 Eating Concern R² 0.231				
Restraint Behaviour						
Dehumanization composite index → IBS (a)	Direct	0.595	0.136	4.365	<.001	[0.336, 0.883]
IBS → Restraint (b)	Direct	0.428	0.204	2.099	.036	[0.067, 0.863]
Dehumanization composite index → Restraint (c’)	Direct	0.001	0.223	0.006	.996	[-0.390, 0.508]
Dehumanization composite index → Restraint	Indirect	0.254	0.135	1.887	.059	[0.050, 0.581]
Dehumanization composite index → Restraint	Total	0.256	0.187	1.365	.172	[-0.069, 0.675]
	Explained variance	IBS R² 0.294 Restraint behaviour R² 0.173				

N, 42. Coefficients are standardized β regression weights. SE, standard error; IBS is the mediator in all models. Indirect effects tested using bias-corrected and accelerated (BCa) bootstrap confidence intervals based on 5,000 resamples. These models are perfectly saturated (df, 0), producing perfect fit indices by mathematical necessity: χ²(0), 0.00, CFI, 1.000; TLI, 1.000; RMSEA, 0.000; SRMR, 0.000. These values do not indicate strong model validation but reflect model saturation.

### 3.4 Moderated mediation analyses

**Figure 3:**
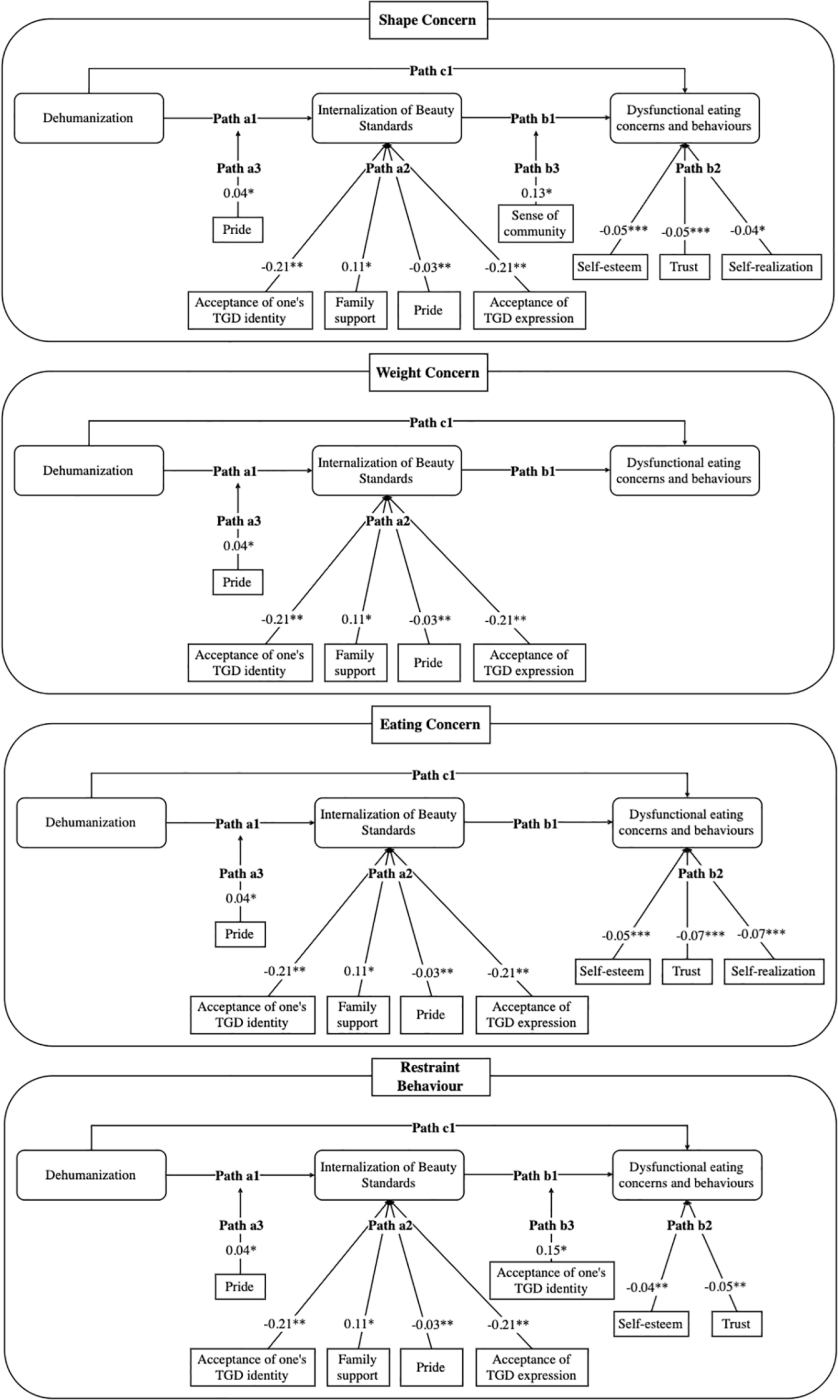
Moderated mediation models. p < .05*, < .01**, < .001***.

In all models, dehumanization composite index significantly predicted IBS, which was strongly associated with shape, weight, eating concerns, and restraint behaviour. TGD Pride, acceptance of TGD identity, and acceptance of gender expression directly predicted lower IBS (Path A2) in all models, while family support predicted higher IBS in all models. TGD Pride also intensified the dehumanization-IBS pathway (Path A3: b = 0.04*, 95% CI [0.00, 0.07]). Resilience factors exerted negative direct effects on outcomes (Path B2): self-esteem, trust, and self-realization for shape and eating concern; and self-esteem and trust for restraint behaviour. The IBS-outcome pathway (Path B3) was strengthened by sense of community in the shape model (b = 0.13*, 95% CI [0.01, 0.24]) and by acceptance of TGD identity in the restraint model (b = 0.15*, 95% CI [0.02, 0.28]) (see **Figure 3**).

Conditional indirect effects indicated that, across moderators, the mediating role of IBS was generally strongest at median levels. Indirect effects also peaked at high levels of TGD pride, community connection (all models), family support (shape and weight models), and acceptance of one’s TGD identity (weight model). In contrast, indirect effects were strongest at low levels of support from significant others (all models), relationship and social support (shape, weight, and restraint models) and sense of community (weight model) (see **Supplementary Figure 1**).

Across models, explained variance for IBS ranged from 0.16 to 0.27. For outcomes, explained variance was 0.32-0.43 for shape concern, 0.69-0.70 for weight concern, 0.18-0.31 for eating concern, and 0.12-0.21% for restraint. Across all 52 models, fit was evaluated using χ², CFI, RMSEA, and SRMR. Given the near-saturated nature of these models (df = 1), the chi-square test was used as the primary fit index, as RMSEA estimates are known to be unstable with very low degrees of freedom. The large majority of models showed acceptable fit, with non-significant chi-square tests (p = .318-.913), RMSEA = .000, CFI = 1.000, and SRMR ≤.011. Three moderators showed suboptimal fit across all four outcomes. Models involving spirituality showed poor fit (RMSEA = .195, χ²(1) = 5.62-5.66, p = .017-.018, CFI = .832-.968), likely attributable to the severe non-normal distribution of this variable (skewness = 2.11, kurtosis = 4.76); Given these distributional violations and poor model fit, spirituality findings are not interpreted as part of the results. Models involving gender expression acceptance showed elevated RMSEA values (RMSEA = .116-.123, CFI = .952-.974), though associated chi-square tests were non-significant (p = .092-.101), indicating acceptable fit by the primary criterion. Models involving self-esteem showed marginally elevated RMSEA values (RMSEA = .059-.061, CFI = .987-.994) with non-significant chi-square tests (p = .227-.232), and are considered interpretable. Full results are reported in **Supplementary Table 2**.

## 4 Discussion

### 4.1 Differential sexualization, identity affirmation, and body related stressors across TGD subgroups

Multiple regression analyses examining associations between sociodemographic variables and psychological outcomes (**Table 3**) revealed several patterns. Experiences of body evaluation over the past year were significantly higher among transgender women than among transgender men and GD xcindividuals. For lifetime experiences of body evaluation and lifetime exposure to explicit sexual advances, significant differences emerged only for GD participants. This pattern may reflect that most participants (82%) had initiated GA more than one year earlier. In Western context, and particularly in Italy, feminine-coded bodies are highly sexualized, a process that may intensify during puberty. For transgender women, socialized as male at birth, one possibility is that such attention may appear later, potentially increasing during GA as their appearance feminizes. In contrast, transgender men may experience early sexualization that may diminish during GA, whereas GD individuals may face persistent evaluation due to nonconformity to binary norms. GD participants also reported significantly higher levels of acceptance of TGD expression than transgender women. However, they also experienced higher non-affirmation of TGD identity. This apparent paradox may reflect the social positioning of GD individuals: they are typically less invested in “passing” within a binary gender, yet encounter persistent external discrimination (56, 57). Transgender men reported lower pride but higher support from significant others than transgender women; this may reflect different social positions. Transgender men’s relative invisibility in media and public discourse may limit opportunities for collective pride, whereas transgender women’s greater visibility and scrutiny, despite heightened discrimination risk, may also foster stronger community ties and collective pride. Moreover, testosterone often produces more visible changes than estrogen, enabling faster cisnormative passing, which can facilitate assimilation and reduce sustained engagement in TGD-specific pride and belonging. Finally, transgender men may experience greater social acceptance in intimate relationships than transgender women, who face compounded stigma at the intersection of transmisogyny.

Time since GA appeared protective: longer duration correlated with lower non-affirmation, internalized transphobia, and concealment, and higher self-esteem, trust and self-realization. Shorter GA was associated with greater IBS athletic ideals, body shape, and weight concerns. Clinically, these results suggest the need to ensure and expand access to GA pathways by removing systemic barriers, as affirmation itself may protect psychological well-being.

Higher BMI predicted lower support from significant others, greater non-affirmation, intensified social-media appearance pressure, and elevated body shape concerns, consistent with dual marginalization from weight stigma and gender minority stress (58). In addition, limited access to gender-affirming surgeries, often constrained by BMI requirements, can further heighten psychological distress and body dysphoria. Conversely, lower BMI related to diminished partner support and reduced self-esteem, consistent with evidence linking body dissatisfaction and low self-esteem among TGD groups (59).

### 4.2 The ambivalent role of resilience factors in risk pathways toward disordered eating

Exploratory moderated mediation analyses (**Supplementary Table 2**) revealed complex patterns that may suggest both protective and risk-amplifying effects. TGD pride, TGD identity acceptance, and expression were associated with lower IBS across all four path analyses, independently of dehumanization composite index, suggesting these factors may directly reduce vulnerability to IBS. Self-esteem and trust were associated with lower outcomes in the shape concern, eating concern, and restraint models, reducing vulnerability independently of both dehumanization composite index and IBS. Similarly, self-realization appear to moderate body shape and eating concerns.

However, some resilience factors also appeared to show ambivalent patterns. Across moderated mediation models, TGD pride was simultaneously protective and potentially exposing: it was associated with lower IBS but appeared to amplify the dehumanization composite index-IBS pathway. These exploratory findings suggest that, although pride may be protective, it may not fully buffer against dehumanization and could potentially intensify its salience via increased visibility to objectification and microaggressions. Prior work is consistent with this potential ambivalent role, showing enhanced body appreciation (60) but greater distress in stigmatizing contexts (61).

In the restraint behaviour model, individuals who felt more accepted for their identity appeared less likely to engage in restrictive eating behaviours. However, at higher levels of acceptance, the IBS-restriction pathway appeared strengthened. One potential interpretation (62) is that acceptance may highten motivation to align embodiment with gendered norms, potentially intensifying body surveillance and restriction. Thus, identity acceptance may not function uniformly as protective.

In the shape concern models, stronger TGD community belonging appeared to strengthen the IBS-outcome association. Although TGD communities provide support and recognition, they are not free of aesthetic norms. Online and offline spaces may circulate idealized, “passable” trans bodies that mirror cisnormative social media standards. These intracommunity ideals can reproduce dominant beauty norms and may heighten pressures for conformity. This exploratory pattern is consistent with intraminority community stress observed among sexual minority men and may be especially relevant for non-binary individuals, whose bodies may fall outside dominant ideals even within community spaces.

Finally, family support appeared to show a positive association with IBS across models. This may reflect well-intentioned but appearance-focused affirmation (e.g., “passing” or transition-related compliments) or family anxieties about social acceptance expressed through body-focused comments. These findings suggest that family support may not uniformly buffer IBS and could potentially reinforce beauty norms linked to eating disorder risk.

### 4.3 Clinical implications

These exploratory findings, may carry several implications for clinical practice with TGD people. Clinicians should systematically explore community-specific appearance norms across online and offline contexts and how these shape body-related distress. They should help clients distinguish gender affirmation motives rooted in authentic expression from those driven by cisnormative aesthetic pressures or anticipated acceptance. Given emerging evidence that gender expression dimensions such as masculinity and femininity may differentially influence eating behaviours, with masculine identification linked to cognitive restraint and feminine identification associated with emotional and uncontrolled eating (63), interventions should address how internalized gender norms shape coping strategies and body experiences. Interventions should target media literacy regarding how online spaces reproduce restrictive beauty standards and reduce appearance-based social comparison. Finally, while community connection and identity pride function as resilience resources, therapeutic approaches should remain attuned to contexts where these factors may inadvertently expose clients to hypergendered aesthetic norms, tailoring interventions to individual sociocultural positioning rather than applying uniform assumptions about protective effects.

### 4.4 Limitations and future directions

This study has several limitations. First, the cross-sectional design precludes causal inference. Future studies should employ longitudinal designs to establish temporal precedence and clarify directionality of effects. Second, the exclusively quantitative approach limits our understanding of the mechanisms underlying these associations. Qualitative methods would enrich interpretation by capturing how TGD individuals subjectively experience community norms, navigate appearance pressures, and interpret body ideals encountered in online and offline spaces. Third, the sample was relatively homogeneous, highlighting the need for more intersectional approaches. Moreover, these findings emerge from an Italian/Western sample where TGD community structures, beauty standards, and minority stress contexts may differ substantially from non-Western settings. Replication across diverse cultural contexts is essential before generalizing these patterns. Fourth, although the findings provide preliminary support for applying the PMD to GD individuals, replication in larger and more diverse samples is needed, findings for this group should be considered preliminary. Fifth, participants were recruited both through the clinic (Regional Reference Center for Gender Incongruence) and via other channels (social media, community organizations, and snowball sampling), though precise proportions per recruitment source were not systematically tracked. This sampling strategy may overrepresent individuals with higher community engagement and greater access to healthcare. Sixth, we did not test measurement invariance across gender identity subgroups; resilience factor scales and eating-related measures may function differently for transgender women, transgender men, and gender-diverse individuals. Due to multiple comparisons (13 moderators × 4 outcomes), some significant findings may reflect Type I error. Results should be considered exploratory pending replication in independent samples. Common-method variance, arising from the use of self-report measures administered in a single session, may inflate associations between constructs. Additionally, IBS and EDE-Q subscales assess conceptually related body- and appearance-focused concerns, which may contribute to shared variance beyond true predictive relationships.

## 5 Conclusion

This study demonstrates that several psychosocial factors, including self-esteem, TGD pride, identity and expression acceptance, interpersonal trust, and self-realization, serve as important buffers against body concerns and disordered eating. However, their effects were not uniformly protective. Our findings indicate that within stigmatizing hetero-cisnormative environments, strong identity or community investment may paradoxically intensify vulnerability when beauty ideals are reproduced or reinforced, including within intracommunity spaces.

These dynamics underscore the need for clinicians and researchers to attend carefully to the contextual conditions under which resilience factors may become double-edged, and to tailor interventions to the specific sociocultural environments shaping TGD embodiment. In addition, by testing the PMD among GD participants, we showed that GD individuals are equally vulnerable to dehumanization and disordered-eating behaviours to those of binary-identified transgender groups.

## Data Availability

The raw data supporting the conclusions of this article will be made available by the authors, without undue reservation.
