# Supplementary material for: Context matters: reconsidering resilience factors in dehumanization pathways to dysfunctional eating among transgender and gender diverse individuals
Source: Front Psychiatry. 2026 Mar 23;17:1788398. doi: 10.3389/fpsyt.2026.1788398 (PMC13052445; doi:10.3389/fpsyt.2026.1788398)
Supplement: Supplementary file 1 [file SupplementaryFile1.docx]

***Supplementary materials***

**Table S1. Mediation analysis: effects of dehumanization** composite index **on eating disorder symptoms via IBS**

| **Path** | **Effect** | **β** | **SE** | **z** | **p** | **95% BCa CI** |
| --- | --- | --- | --- | --- | --- | --- |
| **Shape Concern** | | | | | | |
| Dehumanization composite index → IBS (a) | Direct | 0.282 | 0.109 | 2.579 | .010 | [0.071, 0.499] |
| IBS → Shape Concern (b) | Direct | 0.377 | 0.083 | 4.528 | <.001 | [0.203, 0.531] |
| Dehumanization composite index → Shape Concern (c′) | Direct | 0.197 | 0.098 | 1.998 | .046 | [0.001, 0.397] |
| Dehumanization composite index → Shape Concern | Indirect | 0.106 | 0.049 | 2.179 | .029 | [0.029, 0.225] |
| Dehumanization composite index → Shape Concern | Total | 0.303 | 0.105 | 2.878 | .004 | [0.099, 0.516] |
|  | Explained variance | IBS R² 0.157;  Shape Concern R² 0.318 | | | | |
| **Weight Concern** | | | | | | |
| Dehumanization composite index → IBS (a) | Direct | 0.282 | 0.109 | 2.574 | .010 | [0.070, 0.499] |
| IBS → Weight Concern (b) | Direct | 0.747 | 0.067 | 11.232 | <.001 | [0.592, 0.859] |
| Dehumanization composite index → Weight Concern (c′) | Direct | 0.180 | 0.062 | 2.902 | .004 | [0.068, 0.313] |
| Dehumanization composite index → Weight Concern | Indirect | 0.210 | 0.085 | 2.474 | .013 | [0.055, 0.393] |
| Dehumanization composite index → Weight Concern | Total | 0.390 | 0.108 | 3.613 | <.001 | [0.177, 0.608] |
|  | Explained variance | IBS R² 0.157;  Weight Concern R² 0.688 | | | | |
| **Eating Concern** | | | | | | |
| Dehumanization composite index → IBS (a) | Direct | 0.287 | 0.108 | 2.644 | .008 | [0.078, 0.503] |
| IBS → Eating Concern (b) | Direct | 0.240 | 0.092 | 2.601 | .009 | [0.059, 0.418] |
| Dehumanization composite index → Eating Concern (c′) | Direct | 0.286 | 0.101 | 2.844 | .004 | [0.077, 0.476] |
| Dehumanization composite index → Eating Concern | Indirect | 0.069 | 0.040 | 1.736 | .083 | [0.013, 0.175] |
| Dehumanization composite index → Eating Concern | Total | 0.355 | 0.103 | 3.454 | <.001 | [0.145, 0.553] |
|  | Explained variance | IBS R² 0.159;  Eating Concern R² 0.172 | | | | |
| **Restraint Behaviour** | | | | | | |
| Dehumanization composite index → IBS (a) | Direct | 0.283 | 0.109 | 2.589 | .010 | [0.073, 0.501] |
| IBS → Restraint (b) | Direct | 0.288 | 0.091 | 3.176 | .001 | [0.099, 0.459] |
| Dehumanization composite index → Restraint (c′) | Direct | 0.111 | 0.112 | 0.995 | .320 | [-0.107, 0.336] |
| Dehumanization composite index → Restraint | Indirect | 0.082 | 0.043 | 1.911 | .056 | [0.019, 0.190] |
| Dehumanization composite index → Restraint | Total | 0.193 | 0.113 | 1.706 | .088 | [-0.032, 0.416] |
|  | Explained variance | IBS R² 0.157;  Restraint Behaviour R² 0.111 | | | | |

*Note.* N = 122. Coefficients are standardized β regression weights. IBS = Internalization of Beauty Standards (mediator in all models). Dehumanization is a composite measure of discrimination and objectification experiences. Indirect effects tested using bias-corrected and accelerated (BCa) bootstrap confidence intervals based on 5,000 resamples. These models are perfectly saturated (df = 0), producing perfect fit indices by mathematical necessity: χ²(0) = 0.00, CFI = 1.000, TLI = 1.000, RMSEA = 0.000, SRMR = 0.000. These values do not indicate strong model validation but reflect model saturation.

**Table S2. Moderated mediation analyses: path coefficients and conditional indirect effects of dehumanization on eating disorder outcomes via IBS**

| **Outcome** | **Moderator** | **a₁**  **b(SE)**  **[95% CI]** | **a₂ b(SE) [95% CI]** | **a₃ b(SE) [95% CI]** | **b₁ b(SE) [95% CI]** | **b₂ b(SE) [95% CI]** | **b₃ b(SE) [95% CI]** | **c₁ b(SE) [95% CI]** | **R² IBS** | **R² Outcome** | **Significant Conditional Indirect Effects b [95% CI]** |
| --- | --- | --- | --- | --- | --- | --- | --- | --- | --- | --- | --- |
| **Shape Concern** | Community Connection | 0.32**(0.12)[0.09, 0.54] | — | — | 0.38***(0.09) [0.19, 0.53] | — | — | — | 0.17 | 0.32 | **Median**: 0.12(0.05) [0.03, 0.24];  **High** (+1SD): 0.16(0.08) [0.03, 0.38] |
|  | Pride | 0.29**(0.11) [0.08, 0.49] | -0.03*(0.01) [-0.06, -0.00] | 0.04*(0.02) [0.00, 0.07] | 0.41***(0.09) [0.22, 0.58] | — | — | — | 0.22 | 0.33 | **Median**: 0.12(0.05) [0.04, 0.25];  **High** (+1SD): 0.22(0.09)[0.08, 0.44] |
|  | Support From Significant Others | 0.29*(0.11) [0.06, 0.50] | — | — | 0.39***(0.09) [0.21, 0.57] | — | — | — | 0.20 | 0.32 | **Low** (−1SD): 0.18(0.10)[0.03, 0.42];  **Median**: 0.09(0.05) [0.01, 0.20] |
|  | Family Support | 0.30**(0.11) [0.09, 0.52] | 0.11*(0.05) [0.01, 0.20] | — | 0.38***(0.09) [0.20, 0.55] | — | — | — | 0.20 | 0.32 | **Median:** 0.12(0.05)[0.03, 0.23];  **High** (+1SD): 0.13(0.07)[0.03, 0.30] |
|  | Friends Support | 0.25*(0.11) [0.04, 0.46] | — | — | 0.36***(0.09) [0.18, 0.53] | — | — | — | 0.18 | 0.34 | **Median**: 0.09(0.05)[0.02, 0.20] |
|  | Self-Esteem | 0.24*(0.11) [0.04, 0.46] | — | — | 0.31***(0.08) [0.15, 0.46] | -0.05***(0.01) [-0.07, -0.03] | — | — | 0.19 | 0.42 | **Median**: 0.07(0.04)[0.01, 0.17] |
|  | Trust | 0.23*(0.11) [0.02, 0.46] | — | — | 0.33***(0.08) [0.17, 0.48] | -0.05***(0.01) [-0.08, -0.03] | — | — | 0.18 | 0.43 | **Median**: 0.08(0.04)[0.01, 0.19] |
|  | Spirituality_Chs | 0.27*(0.12) [0.04, 0.50] | — | — | 0.36***(0.09) [0.18, 0.53] | — | — | 0.22*(0.10) [0.01, 0.43] | 0.16 | 0.32 | **Median**: 0.10(0.06)[0.01, 0.27] |
|  | Self-Realization | 0.25*(0.11) [0.05, 0.49] | — | — | 0.35***(0.09) [0.17, 0.51] | -0.04*(0.02) [-0.08, -0.01] | — | — | 0.17 | 0.36 | **Median**: 0.09(0.05)[0.02, 0.21] |
|  | Relationship And Social Support | 0.27*(0.11) [0.06, 0.49] | — | — | 0.37***(0.09) [0.20, 0.54] | — | — | — | 0.17 | 0.32 | **Low** (−1SD): 0.10(0.08)[0.00, 0.32];  **Median**: 0.10(0.05)[0.02, 0.22] |
|  | Acceptance Of One’s Gender Identity | 0.28*(0.12) [0.04, 0.52] | -0.21**(0.06) [-0.32, -0.07] | — | 0.34***(0.10) [0.14, 0.53] | — | — | — | 0.27 | 0.35 | **Median**: 0.10(0.05)[0.02, 0.24] |
|  | Acceptance Of One’s Gender Expression | 0.25*(0.11) [0.04, 0.49] | -0.21**(0.07) [-0.33, -0.07] | — | 0.33***(0.10) [0.13, 0.52] | — | — | 0.23*(0.11) [0.01, 0.45] | 0.25 | 0.34 | **Median**: 0.08(0.05)[0.01, 0.21] |
|  | Sense Of Community | 0.32**(0.12) [0.09, 0.54] | — | — | 0.36***(0.08) [0.19, 0.52] | — | 0.13*(0.06) [0.01, 0.24] | — | 0.18 | 0.34 | **Median**: 0.12(0.05)[0.03, 0.24] |
| **Weight Concern** | Community Connection | 0.32**(0.12) [0.09, 0.54] | — | — | 0.74***(0.07) [0.59, 0.86] | — | — | 0.20**(0.07) [0.07, 0.35] | 0.17 | 0.70 | **Median** : 0.24(0.09)[0.06, 0.42];  **High** (+1SD): 0.26(0.12)[0.05, 0.53] |
|  | Pride | 0.29**(0.11) [0.08, 0.49] | -0.03*(0.01) [-0.06, -0.00] | 0.04*(0.02) [0.00, 0.07] | 0.73***(0.07) [0.56, 0.85] | — | — | 0.20**(0.07) [0.07, 0.34] | 0.22 | 0.70 | **Median**: 0.22(0.08)[0.07, 0.39];  **High** (+1SD): 0.34(0.10)[0.16, 0.57] |
|  | Support From Significant Others | 0.29*(0.11) [0.06, 0.50] | — | — | 0.77***(0.07) [0.61, 0.88] | — | — | 0.17**(0.06) [0.06, 0.31] | 0.20 | 0.69 | **Low** (−1SD): 0.37(0.15)[0.10, 0.68];  **Median**: 0.17(0.09)[0.00, 0.34] |
|  | Family Support | 0.30**(0.11) [0.09, 0.52] | 0.11*(0.05) [0.01, 0.20] | — | 0.76***(0.07) [0.60, 0.88] | — | — | 0.17**(0.06) [0.06, 0.30] | 0.20 | 0.69 | **Median**: 0.24(0.09)[0.08, 0.42];  **High** (+1SD): 0.27(0.11)[0.06, 0.50] |
|  | Friends Support | 0.25*(0.11) [0.04, 0.46] | — | — | 0.75***(0.07) [0.59, 0.87] | — | — | 0.18**(0.06) [0.06, 0.31] | 0.18 | 0.70 | **Median**: 0.18(0.08)[0.03, 0.35] |
|  | Self-Esteem | 0.24*(0.11) [0.04, 0.46] | — | — | 0.75***(0.07) [0.59, 0.87] | — | — | 0.18**(0.06) [0.05, 0.30] | 0.19 | 0.71 | **Median**: 0.18(0.08)[0.03, 0.36] |
|  | Trust | 0.23*(0.11) [0.01, 0.46] | — | — | 0.74***(0.07) [0.58, 0.86] | — | — | 0.18**(0.06) [0.06, 0.31] | 0.18 | 0.70 | **Median**: 0.18(0.09)[0.02, 0.36] |
|  | Spirituality_Chs | 0.27*(0.12) [0.04, 0.50] | — | — | 0.75***(0.07) [0.59, 0.86] | — | — | 0.20**(0.07) [0.08, 0.35] | 0.16 | 0.69 | **Median**: 0.20(0.11)[0.00, 0.43];  **High** (+1SD): 0.20(0.10)[0.03, 0.43] |
|  | Self-Realization | 0.25*(0.11) [0.05, 0.49] | — | — | 0.75***(0.07) [0.58, 0.87] | — | — | 0.19**(0.06) [0.08, 0.33] | 0.17 | 0.69 | **Median**: 0.19(0.09)[0.04, 0.39] |
|  | Relationship And Social Support | 0.27*(0.11) [0.06, 0.49] | — | — | 0.75***(0.07) [0.59, 0.87] | — | — | 0.18**(0.06) [0.06, 0.32] | 0.17 | 0.69 | **Low** (−1SD): 0.23(0.15)[0.00, 0.56];  **Median**: 0.20(0.09)[0.05, 0.38] |
|  | Acceptance Of One’s Gender Identity | 0.28*(0.12) [0.05, 0.52] | -0.21**(0.06) [-0.32, -0.07] | — | 0.73***(0.07) [0.56, 0.86] | — | — | 0.19**(0.07) [0.06, 0.34] | 0.27 | 0.69 | **Median**: 0.20(0.09)[0.04, 0.38] |
|  | Acceptance Of One’s Gender Expression | 0.25*(0.11) [0.04, 0.49] | -0.21**(0.07) [-0.33, -0.08] | — | 0.75***(0.07) [0.59, 0.87] | — | — | 0.21**(0.07) [0.08, 0.36] | 0.25 | 0.69 | **Median**: 0.19(0.09)[0.03, 0.38];  **High** (+1SD): 0.21(0.12)[0.00, 0.47] |
|  | Sense Of Community | 0.31**(0.12) [0.08, 0.55] | — | — | 0.73***(0.07) [0.57, 0.85] | — | — | 0.22**(0.07) [0.09, 0.37] | 0.18 | 0.70 | **Low** (−1SD): 0.28(0.13)[0.06, 0.59];  **Median**: 0.22(0.09)[0.06, 0.41] |
| **Eating Concern** | Community Connection | 0.32**(0.12) [0.09, 0.55] | — | — | 0.25*(0.10) [0.06, 0.42] | — | — | 0.27*(0.12) [0.02, 0.48] | 0.17 | 0.18 | **Median** : 0.08(0.05)[0.01, 0.20];  **High** (+1SD): 0.10(0.07)[0.01, 0.29] |
|  | Pride | 0.29**(0.10) [0.09, 0.50] | -0.03*(0.01) [-0.06, -0.00] | 0.04*(0.02) [0.00, 0.07] | 0.27**(0.10) [0.07, 0.46] | — | — | 0.28**(0.11) [0.06, 0.48] | 0.22 | 0.19 | **Median**: 0.08(0.04)[0.02, 0.19];  **High** (+1SD): 0.15(0.09)[0.01, 0.35] |
|  | Support From Significant Others | 0.29**(0.11) [0.07, 0.50] | — | — | 0.25*(0.10) [0.05, 0.44] | — | — | 0.28**(0.11) [0.06, 0.47] | 0.20 | 0.18 | **Low** (−1SD): 0.16(0.09)[0.02, 0.40];  **Median**: 0.05(0.04)[0.00, 0.15] |
|  | Family Support | 0.30**(0.11) [0.10, 0.52] | 0.11*(0.05) [0.01, 0.20] | — | 0.27**(0.09) [0.08, 0.45] | — | — | 0.27**(0.10) [0.06, 0.47] | 0.20 | 0.20 | **Median**: 0.08(0.04)[0.02, 0.19] |
|  | Friends Support | 0.25*(0.11) [0.04, 0.46] | — | — | 0.21*(0.10) [0.02, 0.40] | — | — | 0.27*(0.10) [0.06, 0.47] | 0.18 | 0.22 | **Median**: 0.05(0.03)[0.00, 0.14] |
|  | Self-Esteem | 0.24*(0.11) [0.04, 0.46] | — | — | 0.18†(0.09) [-0.00, 0.37] | -0.05***(0.01) [-0.07, -0.02] | — | 0.23*(0.10) [0.02, 0.42] | 0.19 | 0.26 | **Median**: 0.04(0.03)[0.00, 0.13] |
|  | Trust | 0.23*(0.11) [0.02, 0.47] | — | — | 0.18*(0.09) [0.01, 0.36] | -0.07***(0.02) [-0.10, -0.03] | — | — | 0.18 | 0.31 | **Median**: 0.04(0.03)[0.00, 0.13] |
|  | Spirituality_Chs | 0.27*(0.12) [0.05, 0.51] | — | — | 0.22*(0.09) [0.04, 0.41] | — | — | 0.33**(0.10) [0.11, 0.52] | 0.17 | 0.20 | **Low** (−1SD): 0.08(0.06)[0.00, 0.25];  **Median**: 0.08(0.05)[0.01, 0.23] |
|  | Self-Realization | 0.25*(0.11) [0.05, 0.49] | — | — | 0.20*(0.09) [0.02, 0.37] | -0.07***(0.02) [-0.11, -0.03] | — | — | 0.17 | 0.27 | **Median**: 0.05(0.03)[0.01, 0.16] |
|  | Relationship And Social Support | 0.28*(0.11) [0.06, 0.50] | — | — | 0.23*(0.09) [0.05, 0.42] | — | — | 0.28**(0.11) [0.07, 0.48] | 0.17 | 0.18 | **Median**: 0.06(0.04)[0.01, 0.17] |
|  | Acceptance Of One’s Gender Identity | 0.28*(0.12) [0.05, 0.52] | -0.21***(0.06) [-0.32, -0.08] | — | 0.24*(0.11) [0.03, 0.45] | — | — | 0.27*(0.11) [0.03, 0.48] | 0.27 | 0.19 | **Median**: 0.08(0.05)[0.01, 0.20] |
|  | Acceptance Of One’s Gender Expression | 0.26*(0.11) [0.05, 0.49] | -0.21**(0.07) [-0.33, -0.08] | — | 0.20†(0.11) [-0.02, 0.40] | — | — | 0.30**(0.11) [0.07, 0.51] | 0.25 | 0.19 | **Median**: 0.05(0.04)[0.00, 0.16] |
|  | Sense Of Community | 0.32**(0.12) [0.09, 0.54] | — | — | 0.22*(0.09) [0.04, 0.41] | — | — | 0.28*(0.11) [0.04, 0.49] | 0.18 | 0.20 | **Median**: 0.08(0.04)[0.01, 0.19] |
| **Restraint behavior** | Community Connection | 0.32**(0.12) [0.09, 0.54] | — | — | 0.29**(0.10) [0.09, 0.46] | — | — | — | 0.17 | 0.12 | **Median** : 0.09(0.05)[0.02, 0.21];  **High** (+1SD): 0.13(0.08)[0.02, 0.34] |
|  | Pride | 0.29**(0.10) [0.08, 0.49] | -0.03*(0.01) [-0.06, -0.00] | 0.04*(0.02) [0.00, 0.07] | 0.33***(0.10) [0.13, 0.52] | — | — | — | 0.22 | 0.13 | **Median**: 0.10(0.05)[0.03, 0.22];  **High** (+1SD): 0.20(0.09)[0.05, 0.40] |
|  | Support From Significant Others | 0.29*(0.11) [0.07, 0.50] | — | — | 0.29**(0.10) [0.10, 0.48] | — | — | — | 0.20 | 0.12 | **Low** (−1SD): 0.13(0.09)[0.01, 0.36];  **Median**: 0.07(0.04)[0.01, 0.17] |
|  | Family Support | 0.30**(0.11) [0.09, 0.52] | 0.11*(0.05) [0.01, 0.20] | — | 0.32***(0.09) [0.13, 0.50] | — | — | — | 0.20 | 0.15 | **Median**: 0.09(0.04)[0.02, 0.21] |
|  | Friends Support | 0.25*(0.11) [0.04, 0.46] | — | — | 0.28**(0.10) [0.08, 0.46] | — | — | — | 0.18 | 0.14 | **Median**: 0.07(0.04)[0.01, 0.17] |
|  | Self-Esteem | 0.24*(0.11) [0.04, 0.46] | — | — | 0.24**(0.09) [0.05, 0.41] | -0.04**(0.01) [-0.07, -0.02] | — | — | 0.19 | 0.20 | **Median**: 0.06(0.03)[0.01, 0.15] |
|  | Trust | 0.23*(0.11) [0.02, 0.47] | — | — | 0.24**(0.09) [0.06, 0.41] | -0.05**(0.02) [-0.09, -0.02] | — | — | 0.18 | 0.21 | **Median**: 0.05(0.03)[0.01, 0.15] |
|  | Spirituality_Chs | 0.27*(0.12) [0.04, 0.51] | — | — | 0.28**(0.10) [0.09, 0.46] | — | — | — | 0.17 | 0.14 | **Median**: 0.08(0.05)[0.01, 0.23] |
|  | Self-Realization | 0.25*(0.11) [0.05, 0.49] | — | — | 0.27**(0.09) [0.08, 0.44] | — | — | — | 0.17 | 0.15 | **Median**: 0.07(0.04)[0.01, 0.18] |
|  | Relationship And Social Support | 0.27*(0.11) [0.06, 0.49] | — | — | 0.28**(0.09) [0.09, 0.46] | — | — | — | 0.17 | 0.13 | **Low** (−1SD): 0.10(0.08)[0.00, 0.31];  **Median**: 0.08(0.04)[0.01, 0.18] |
|  | Acceptance Of One’s Gender Identity | 0.28*(0.12) [0.05, 0.52] | -0.21**(0.06) [-0.32, -0.08] | — | 0.29**(0.11) [0.07, 0.50] | — | 0.15*(0.06) [0.02, 0.28] | — | 0.27 | 0.17 | **Median**: 0.09(0.05)[0.02, 0.23] |
|  | Acceptance Of One’s Gender Expression | 0.25*(0.11) [0.04, 0.49] | -0.21**(0.07) [-0.33, -0.07] | — | 0.24*(0.10) [0.03, 0.44] | — | — | — | 0.25 | 0.18 | **Median**: 0.05(0.04)[0.00, 0.16] |
|  | Sense Of Community | 0.32**(0.12) [0.09, 0.55] | — | — | 0.26**(0.10) [0.07, 0.45] | — | — | — | 0.18 | 0.13 | **Median**: 0.09(0.05)[0.02, 0.20] |

*Note*. N = 122. ; Dehumanization is a composite measure of discrimination and objectification experiences.. IBS = Internalization of Beauty Standards; SE = standard error; CI = bias-corrected bootstrapped 95% confidence interval. Unstandardized coefficients (b) are reported for all paths. a₁ = Dehumanization composite index → IBS; a₂ = Moderator → IBS; a₃ = Dehumanization composite index × Moderator → IBS; b₁ = IBS → Outcome; b₂ = Moderator → Outcome; b₃ = IBS × Moderator → Outcome; c₁ = Dehumanization composite index → Outcome (direct effect). Conditional indirect effects (ab) are reported at low (−1 SD / low score), median, and high (+1 SD / high score) levels of the moderator; only levels with 95% CI excluding zero are reported. Non-significant paths (p ≥ .05) shown as —.. p < .05*, < .01**, < .001***. R² indicates explained variance in IBS and each outcome.

**Figure S1: Key moderating interactions in the dehumanization** composite index**-IBS-disordered eating pathway**


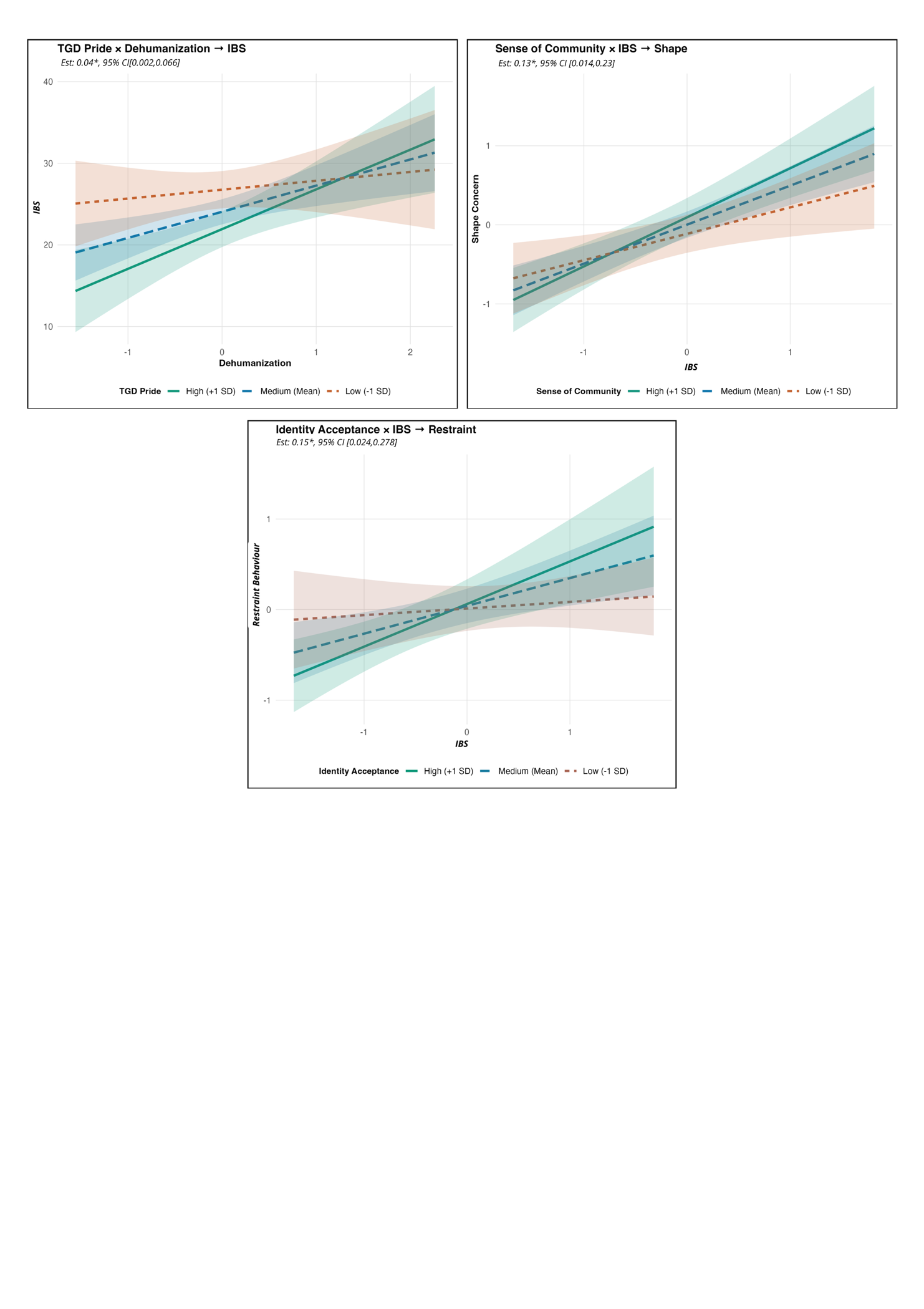


**Supplementary Appendix: template for simple mediation models and moderated mediation models**

All models were estimated using the lavaan package (version 0.6-18) in R (version 4.4.3).

*ESTIMATION SPECIFICATIONS (COMMON TO ALL MODELS):*

- Estimator: Maximum Likelihood (ML)

- Missing data: Full Information Maximum Likelihood (FIML)

- Standard errors: Bootstrap (5,000 resamples)

- Confidence intervals: Bias-corrected and accelerated (BCa) 95% CIs

- Seed for reproducibility: 123

- Exogenous variables: Treated as random (fixed.x = FALSE)

*COVARIATES (INCLUDED IN ALL MODELS):*

- Age (ordinal: 18-24, 25-34, 35-64)

- BMI (mean-centered continuous)

- Time since gender affirmation (ordinal: <1, 1-2, 3-5, 6-10, >10 years)

- Gender identity (dummy coded: Trans Men, Gender Diverse; reference = Trans Women)

*VARIABLE DEFINITIONS:*

- DEHUMANIZATION_composite: Mean of standardized discrimination (D_GMSR) and body evaluation past year (BE_PASTYEAR_ISOS)

- INTERNALIZATION_GENERAL_SATAQ_z: Standardized internalization of beauty standards (mediator)

- Outcomes: SHAPE_CONCERN_EDEQ_z, WEIGHT_CONCERN_EDEQ_z, EATING_CONCERN_EDEQ_z, RESTRAINT_EDEQ_z

- Moderators: 13 resilience factors (mean-centered before computing interaction terms)

**TEMPLATE 1: SIMPLE MEDIATION MODEL**

This model tests the indirect effect of dehumanization composite index on eating disorder outcomes

through internalization of beauty standards, controlling for covariates.

model <- '

# Path a: Dehumanization → Internalization (mediator)

INTERNALIZATION_GENERAL_SATAQ_z ~ a*DEHUMANIZATION_composite +

age_numeric + BMI_centered +

tempo_numeric + gender_men + gender_diverse

# Paths b and c': Mediator and predictor → Outcome

SHAPE_CONCERN_EDEQ_z ~ b*INTERNALIZATION_GENERAL_SATAQ_z +

c*DEHUMANIZATION_composite +

age_numeric + BMI_centered +

tempo_numeric + gender_men + gender_diverse

# Defined parameters

indirect := a * b # Indirect effect (mediation)

direct := c # Direct effect (c' path)

total := c + (a * b) # Total effect

'

fit <- sem(model,

data = df,

estimator = "ML",

missing = "fiml",

fixed.x = FALSE,

se = "bootstrap",

bootstrap = 5000,

iseed = 123,

parallel = "no",

warn = FALSE)

**TEMPLATE 2: MODERATED MEDIATION MODEL**

This model tests whether resilience factors moderate the mediation pathway at:

- Path A: Dehumanization → Internalization (first-stage moderation)

- Path B: Internalization → Outcome (second-stage moderation)

- Path C: Dehumanization → Outcome (direct effect moderation)

EXAMPLE: Self-Esteem as Moderator of Shape Concern

*VARIABLE PREPARATION:*

# Mean-center moderator

df$ROSENBERG_c <- scale(df$ROSENBERG, center = TRUE, scale = FALSE)

# Create interaction terms

df$DISUM_ROSENBERG <- df$DEHUMANIZATION_composite * df$ROSENBERG_c # Path A interaction

df$INT_ROSENBERG <- df$INTERNALIZATION_GENERAL_SATAQ_z * df$ROSENBERG_c # Path B interaction

# Calculate percentiles for conditional effects (16th, 50th, 84th)

# These approximate low (-1 SD), median, and high (+1 SD) levels

percentiles <- quantile(df$ROSENBERG_c, probs = c(0.16, 0.50, 0.84), na.rm = TRUE)

p16 <- percentiles[1] # Low moderator level

p50 <- percentiles[2] # Median moderator level

p84 <- percentiles[3] # High moderator level

*LAVAAN SYNTAX:*

model <- '

# Path A: First-stage moderation (Dehumanization → Internalization)

INTERNALIZATION_GENERAL_SATAQ_z ~ a1*DEHUMANIZATION_composite +

a2*ROSENBERG_c +

a3*DISUM_ROSENBERG +

age_numeric + BMI_centered +

tempo_numeric + gender_men + gender_diverse

# Paths B and C: Second-stage moderation and direct effect moderation

SHAPE_CONCERN_EDEQ_z ~ b1*INTERNALIZATION_GENERAL_SATAQ_z +

b2*ROSENBERG_c +

b3*INT_ROSENBERG +

c1*DEHUMANIZATION_composite +

c2*DISUM_ROSENBERG +

age_numeric + BMI_centered +

tempo_numeric + gender_men + gender_diverse

# Conditional simple slopes for Path A (at low, median, high moderator)

aLow := a1 + a3*(-0.58) # Effect at 16th percentile

aMedian := a1 + a3*(0.00) # Effect at 50th percentile (median)

aHigh := a1 + a3*(0.58) # Effect at 84th percentile

# Conditional simple slopes for Path B (at low, median, high moderator)

bLow := b1 + b3*(-0.58) # Effect at 16th percentile

bMedian := b1 + b3*(0.00) # Effect at 50th percentile (median)

bHigh := b1 + b3*(0.58) # Effect at 84th percentile

# Conditional indirect effects (mediation at different moderator levels)

abLow := aLow * bLow # Indirect effect at low self-esteem

abMedian := aMedian * bMedian # Indirect effect at median self-esteem

abHigh := aHigh * bHigh # Indirect effect at high self-esteem

'

fit <- sem(model,

data = df,

estimator = "ML",

missing = "fiml",

fixed.x = FALSE,

se = "bootstrap",

bootstrap = 5000,

iseed = 123,

parallel = "no",

warn = FALSE)
